# Pedestrian Dead Reckoning Based on Motion Mode Recognition Using a Smartphone

**DOI:** 10.3390/s18061811

**Published:** 2018-06-04

**Authors:** Boyuan Wang, Xuelin Liu, Baoguo Yu, Ruicai Jia, Xingli Gan

**Affiliations:** 1 College of Information and Communication Engineering, Harbin Engineering University, Harbin 150001, China; boyuan@hrbeu.edu.cn; 2 State Key Laboratory of Satellite Navigation System and Equipment Technology, Shijiazhuang 050081, China; yubg@sina.cn (B.Y.); jiaruicai@126.com (R.J.); ganxingli@126.com (X.G.); 3 The 54th Research Institute of China Electronics Technology Group Corporation, Shijiazhuang 050081, China

**Keywords:** indoor localization, motion mode recognition, pedestrian dead reckoning, heading determination, smartphone sensors

## Abstract

This paper presents a pedestrian dead reckoning (PDR) approach based on motion mode recognition using a smartphone. The motion mode consists of pedestrian movement state and phone pose. With the support vector machine (SVM) and the decision tree (DT), the arbitrary combinations of movement state and phone pose can be recognized successfully. In the traditional principal component analysis based (PCA-based) method, the obtained horizontal accelerations in one stride time interval cannot be guaranteed to be horizontal and the pedestrian’s direction vector will be influenced. To solve this problem, we propose a PCA-based method with global accelerations (PCA-GA) to infer pedestrian’s headings. Besides, based on the further analysis of phone poses, an ambiguity elimination method is also developed to calibrate the obtained headings. The results indicate that the recognition accuracy of the combinations of movement states and phone poses can be 92.4%. The 50% and 75% absolute estimation errors of pedestrian’s headings are 5.6° and 9.2°, respectively. This novel PCA-GA based method can achieve higher accuracy than traditional PCA-based method and heading offset method. The localization error can reduce to around 3.5 m in a trajectory of 164 m for different movement states and phone poses.

## 1. Introduction

In recent years, location-based services (LBS) have become essential services for people’s daily work and lives [1]. A great requirement of LBS is needed in many fields, such as emergency security, intelligent warehousing, crowd monitoring, precision marketing, mobile health, virtual reality and other fields [2]. The Global Navigation Satellite System (GNSS) plays an important role in LBS and the various enhancement technologies can make GNSS achieve sub meter positioning accuracy in an open area [3]. However, the performance of GNSS reduces seriously in indoor environment because the availability of navigational satellite signals cannot be guaranteed. According to a U.S. Environmental Protection Agency report, people spend nearly 70% to 90% of their time indoors [4]. Therefore, it is significant to establish an accurate, reliable and real-time indoor localization system to satisfy the indoor localization needs of the public.

In advanced smartphones, more sensors are embedded in the phone and the computing capability of the microprocessor of the phone is increased. Current smartphone technology provides an excellent tool for developing indoor positioning systems (IPS) based on processing signals from Radio Frequency (RF) beacons, signals of opportunity and inertial data [5]. Therefore, the indoor localization based on smartphones has become the mainstream technique. Many technologies have emerged to provide the positioning indoors, they are based on wireless signals (such as Wi-Fi, cellular network, Bluetooth and ultra-wide band), vision sensors (such as laser scanner, monocular and binocular camera) and inertial sensors (such as accelerometer, gyroscope and magnetometer). However, due to the diversity of applications, scenarios, sensors and user requirements, it is difficult to create a universally applicable solution for indoor localization [6].

Wi-Fi chips have been widely applied in various smartphones and other mobile devices. Wi-Fi networks cover many public places, such as office buildings, airports and shopping malls. Therefore, the use of Wi-Fi signals for indoor localization is a reasonable choice. Wi-Fi fingerprint localization is a widely used indoor localization technique, which usually consists of two phases: an off-line phase and an on-line phase [7]. During the off-line phase, the known locations of a certain area are selected and the received signal strength indications (RSSI) from multiple Wi-Fi access points (APs) are recorded at the selected locations. The Wi-Fi fingerprint database is generated from known locations and the corresponding RSSI. During the on-line phase, the RSSI from the unknown locations are matched with that from the known locations in the fingerprint database, thus the user location is estimated. However, changes to the space environment result in an inaccurate positioning result, thus the fingerprint database needs to be updated regularly, which is the main challenge of fingerprint location techniques [8]. The localization techniques based on vision sensors can be divided into two categories. One involves collecting images with a mobile camera and the location of the camera is determined, while the other collects images with a fixed camera and the target location in the image is determined [9]. Large graphic computation is required in vision localization systems, because of the hardware limitations, and although the systems have a high precision in a specific environment, the real-time performance may not be guaranteed. With further improvement of smartphone computing capability, this vision localization technique is expected to be further applied and popularized in indoor localization.

With the development of micro-electromechanical system (MEMS) technology, low cost inertial measurement units (IMUs) such as accelerometers, gyroscopes and magnetometers, etc., have been widely embedded in most smartphones. These low cost IMUs have the advantages of small size, light weight and low power consumption [10]. Pedestrian dead reckoning (PDR) is a localization technique that utilizes IMU data to calculate the pedestrian location. Compared with the localization techniques based on wireless signals and vision sensors, PDR can give an accurate position in a short period of time, its updating speed of the pedestrian location is faster and the power consumption is lower. More importantly, since additional infrastructure assistance is not required, PDR systems are simpler and more autonomous. Existing PDR systems can be divided into mounted-PDR systems based on a specialized device and handheld-PDR systems based on a handheld device. In mounted-PDR systems, the accuracy of the device is higher, and it is mounted to a certain part of the body, such as feet, legs and waist. It is regarded as a body-fixed system and the location of the pedestrian is obtained by the time integral on the signals of accelerometers and gyroscopes. Because of the accumulative error, the localization accuracy of mounted-PDR will decrease with time, thus a Zero Velocity Update (ZUPT) algorithm is used to control the accumulative error [11,12,13]. Since pedestrians’ burden is increased, mounted-PDR is less convenient for ordinary consumers in many scenarios [14]. To get rid of the limitation that the device must be mounted to the body, more flexible handheld-PDR systems have been universally used. Handheld-PDR utilizes handheld mobile devices to obtain the locations and headings of pedestrians, which usually consists of three modules: step detection, step length estimation and heading determination. However, there are still some limitations in the existing techniques, as many localization approaches assume that the heading angle offset remains constant, the heading angle offset is the angle between the direction of smartphone and the direction of pedestrian [15,16,17]. The assumption can be satisfied when pedestrians hold smartphones on the front of the body or when pedestrians are making calls. However, during the localization, the phone pose is arbitrary and the heading offset cannot be guaranteed to be constant. Thus, this paper mainly addresses the issues of motion mode recognition and indoor localization of pedestrians. Our method improves the accuracy and flexibility of PDR system by solving the issues of pedestrians moving in different states and the smartphone holding in different poses. The main contributions of our work are as follows:

1. The motion mode can be divided into two categories: the movement state and the phone pose. The movement state represents the global motion of pedestrian and the phone pose represents the pose of people holding or placing smartphones. The movement state and the phone pose are independent with each other, and they can be combined arbitrarily. In prior works, only few combination modes are considered. Therefore, in this paper, we adequately consider all 16 combination modes, which are generated by four movement states (Walking, Running, Upstairs and Downstairs) and four phone poses (Holding, Calling, Swinging and Pocket). We also analyze the characteristics of accelerometer and gyroscope data in different modes in detail. Their features are extracted at the same time. We choose the support vector machine (SVM) and the decision tree (DT) as our classifier. The classification approach proposed in this paper can accurately recognize any combination mode of the movement state and the phone pose.
2. The accelerometer data are different depending on the various movement states and phone poses. To adapt to the change, we present an adaptive step detection algorithm. The thresholds of valid peak and minimum step interval are adjusted with the consideration of different movement states and phone poses, and adjacent peaks selection mechanism is added to eliminate the influence of false peaks. Therefore, the pedestrian’s steps can be detected accurately with the presented algorithm.
3. We improve the traditional principal component analysis-based (PCA-based) method by developing the PCA-based method with global accelerations (PCA-GA) to infer pedestrian’s headings. We extract a more stable right-vector of pedestrian and avoid the horizontal acceleration errors caused by the change of smartphone’s attitude. To solve the ambiguity problem of the extracted right-vector, we further analyze the signals of accelerometer and gyroscope during pedestrian walking. The analysis includes the orientations of the smartphone carried in the front pocket of trousers, and pedestrian swings the smartphone with the left-hand or the right-hand.

The remainder of this paper is organized as follows: in Section 2, related works are discussed. Section 3 presents the system overview. In Section 4, the pre-processing process is introduced. In Section 5, the movement state and phone pose definition, feature extraction and classification method are illustrated. The PDR algorithm adapted to different motions is described in Section 6, including step detection, step length estimation and heading determination. The experimental results of motion recognition and indoor localization are shown in Section 7. Finally, conclusions and future work are discussed in Section 8.

## 2. Related Works

Mobile intelligent platforms have developed rapidly in recent years. Smartphones, smart-glasses, smart-bracelets and smart-watches have become the main terminals of indoor localization systems. Unlike dedicated navigation devices fixed to the body, the users can use these portable devices at will. Thus, the attitude of the portable device can change in real-time and the relative position between the user and the device is not so stable, which brings more challenges to PDR systems. There are some restrictions on the current handheld-PDR system in many application scenarios. For example, smartphones are restricted to being on the front side of the body stably without changing poses [18,19,20,21], otherwise, the pedestrian’s position cannot be obtained, which greatly increases the limitation of localization and cannot satisfy the portability requirement of pedestrians. Some people have classified the possible motion modes during navigation to assist indoor localization. Ling et al. [22] presented an indoor navigation solution by combining physical behavior recognition with wireless positioning. They compared 27 features extracted from the smartphone sensors and the least squares support vector machine (LS-SVM) classification algorithm is used to detect eight behavior patterns that commonly occur in indoor navigation. The accuracy range of the classifier was from 80.4% to 95.5% with different feature selection, and the recognition behaviors significantly improved the wireless location accuracy. Susi et al. [23] studied the irregular motion, which does not contribute to the pedestrian’s displacement, and the recognition of the irregular motion avoided the erroneous judgement of pedestrian’s steps. Shin et al. [24] defined six kinds of common movements in indoor navigation and utilized an artificial neural network (ANN) for the classifier. In [25], the motions of taking an elevator, and standing or walking on an escalator were taken into consideration. Elhoushi et al. [26] first carried out work about detecting standing or walking on a moving walkway versus normal standing or walking, the classification accuracy ranged from 73.1% to 97.2%. In [27], the finite state machine was utilized to conduct practical tracking of pedestrians, and the transition of the smartphone poses can be detected. To relieve the burden of designing and selecting features, Khoshelham et al. [28] first applied stacked denoising autoencoders for locomotion activity recognition. The proposed method was independent of the expert knowledge and greatly reduced the work of manual feature design, by the selection and combination of various sensors, the results showed that the classification performance by multiple sensors can be better than that by only accelerometers. Gu et al. [29] developed a novel feature, called pressure derivative, which was obtained from the barometer embedded in the smartphone to recognize the motions in the vertical plane. In addition, they added the history information of the motion modes to the classification. ANN, SVM, DT and deep learning method have been adopted for the classification of pedestrian’s movement states and phone pose in different scenarios and applications. However, the movement states and the phone poses can be combined arbitrarily in localization process. In this paper, to achieve a better performance of the combination mode recognition, we select two kinds of classifiers: DT and SVM.

In the PDR system, a slight deviation of the headings will lead to a large error in the final position of the predicted trajectory, thus the determination of pedestrian’s heading is the most important and difficult part in the whole system. Since the phone poses are not constant during the localization, the pedestrian’s heading estimation becomes more complicated. In prior works, accelerometers, gyroscopes and magnetometers are used to obtain the yaw angle of the device, and the yaw angle is regarded as the heading of the pedestrian. Using these methods, the device must always point to the direction of pedestrian movement [18,19,20,21], but this limitation cannot be feasible all the times. Indoor map information and landmarks have been utilized to constrain the pedestrian’s trajectory and improve the localization performance in [30,31,32,33], however, when the trajectory covers more than the coverage of map information and landmarks, the localization accuracy will decrease significantly. Shen et al. [34] proposed an indoor location method, which enhanced the heading estimation of PDR with the received signal strength indicator (RSSI) from the Wi-Fi access points (APs). When a long straight trajectory has been detected by PDR, the headings of PDR can be corrected by the headings derived from the RSSI data. However, the heading correction method is only applicable for the pedestrians’ straight walking. In [27], the heading deviation between different phone poses was estimated and the actual heading was obtained by adding the deviation to the smartphone’s orientation. However, when the smartphone is in the dynamic state, such as swinging or placed in the pocket, the prior information of heading deviation will be incorrect. Kunze [35] developed a PCA-based method to infer the orientation of mobile device carried in a pocket from the acceleration signal. Steinhoff et al. [36] used different PCA-based variants to obtain the user’s motion axis and the acceleration signals were filtered more adequately. Deng et al. [37] projected all the acceleration signals into a reference coordinate system using a related rotation matrix, the acceleration signals in the reference coordinate system were used to extract the motion axis by the PCA. However, in these PCA-based methods, the horizontal accelerations need to be first obtained by the related vertical accelerations, and the smartphone’s attitude varies constantly while the pedestrian is walking. Thus, the obtained horizontal accelerations in one stride time interval cannot be guaranteed to be horizontal and the pedestrian’s direction vector will be influenced by the accelerations in the vertical plane. In this paper, we develop a PCA-based heading determination method with global accelerations (PCA-GA) to infer the pedestrian’s headings, which reduces the errors caused by the calculation of the horizontal accelerations.

## 3. System Overview

**Figure 1:**
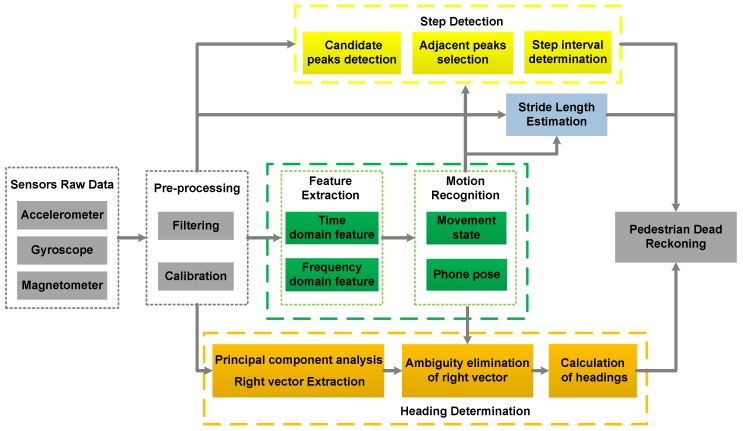
The architecture of the system.

The architecture of the PDR system based on motion mode recognition, which consists of data pre-processing, motion mode recognition and PDR, is shown in Figure 1. The raw data from smartphone sensors contains random noise, and the magnetometer data can be disturbed by the local magnetic environment. Thus, the raw data must be filtered and calibrated. The time-domain features and the frequency-domain features are extracted from the pre-processed data, which are the input of the classifier. The SVM and DT are employed for the classification of the combination modes, according to the movement states recognized by the SVM, the DT distinguishes between different phone poses. The parameters of step detection, stride length estimation and heading determination are adjusted based on the results of the classifier, and the locations of the pedestrian are updated by the equation of PDR. The details of the pedestrian localization system will be further discussed in the following sections.

## 4. Data Pre-Processing

Because of the low measuring accuracy of the sensors embedded in the smartphone, there is a lot of noise in the raw signals from the sensors. Therefore, a pre-processing process should be adopted to eliminate the noise and errors before processing the motion mode recognition and pedestrian localization.

### 4.1. Low-Pass Filtering and Smoothing

**Figure 2:**
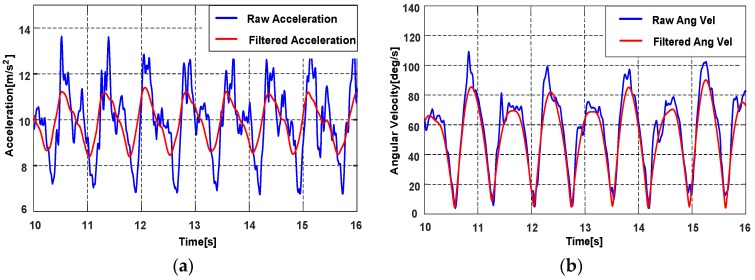
The raw and filtered signal from accelerometer and gyroscope. (**a**) The raw and filtered accelerations; (**b**) The raw and filtered angular velocities.

Based on the analysis of the sensor signal in different motion modes, we found that most energies of the signals are below 15 Hz, so a low-pass filter with a 15 Hz cut-off frequency is adopted to reject the high frequency noise. The signals after the low-pass filter are smoothed again by a moving average filter. As shown in Figure 2, compared with the raw signal, the filtered signal is less noisy and smoother, thus the motions associated to the pedestrian can be reflected more clearly.

### 4.2. Magnetometer Calibration

**Figure 3:**
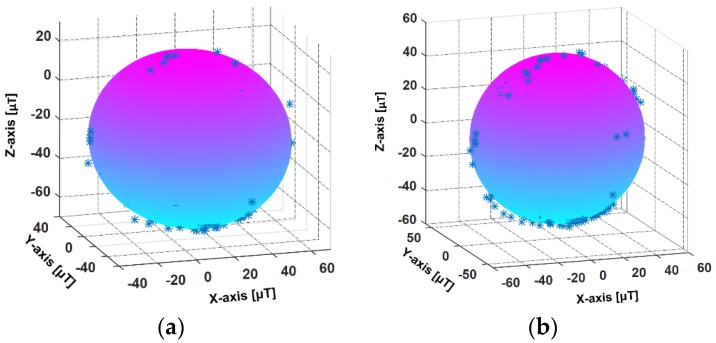
The calibration of the magnetometer. (**a**) The ellipsoid fitting model from the raw magnetometer data; (**b**) The ellipsoid fitting model from the calibrated magnetometer data.

The smartphone magnetometer has large measurement errors and can be easily disturbed by the local magnetic environment. The interferences include hard-iron interference and soft-iron interference. The hard-iron interference is mainly caused by permanent magnet materials in the surrounding environment and the bias is constant. The soft-iron interference is the magnetic field distortion produced by the magnetized matter near the sensor. Since the magnetometer data are used to estimate the headings of pedestrian, magnetometer calibration is necessary. In this paper, the least square fitting ellipsoid method [38] is adopted to calibrate the magnetometer signal. Figure 3a,b represent the ellipsoid fitting models from the raw and calibrated magnetometer data, respectively. In the real environment, the magnetic interference changes with the surrounding environment, thus it is necessary to calibrate the magnetometer in different places. In our works, to guarantee the accuracy of magnetometer data, the magnetometer calibration and the localization experiment are executed in an approximate laboratory environment.

## 5. Motion Mode Recognition

The motion of the pedestrian and smartphone is arbitrary in the navigation process, so the accelerometer and gyroscope signals also have various formats. Therefore, different motion modes should be recognized to assist the handheld-PDR system. In this paper, the accelerations and angular velocities are utilized for the motion recognition, the classification algorithm includes movement state and phone pose definition, feature extraction and the classifier.

### 5.1. Device Coordinate System

**Figure 4:**
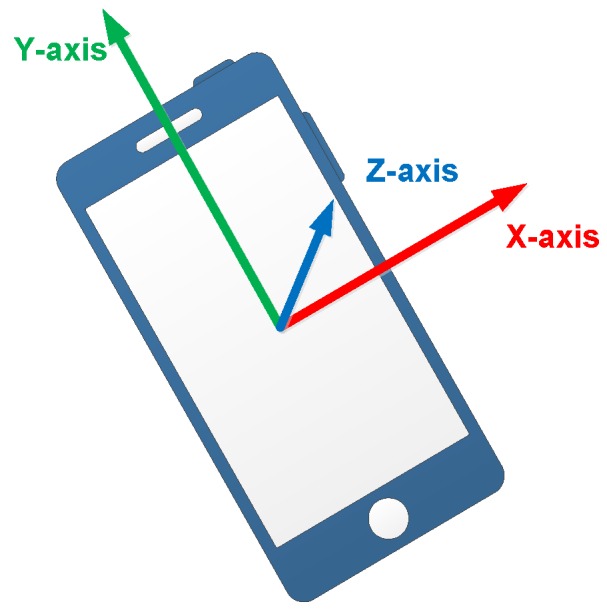
The device coordinate system.

To illustrate the following process of mode recognition and localization, the device coordinate system is introduced first. The coordinate system of the embedded sensors is the same as the device coordinate system, as shown in Figure 4. We place the smartphone horizontally, so the screen center is the coordinate origin, X-axis is parallel to the short side of smartphone and the positive axis is pointing to right on the horizontal plane. Y-axis is parallel to the long side of smartphone and the positive axis is pointing forward on the horizontal plane. The positive direction of Z-axis is pointing upwards and perpendicular to the screen plane.

### 5.2. Movement State and Phone Pose Definition

**Figure 5:**
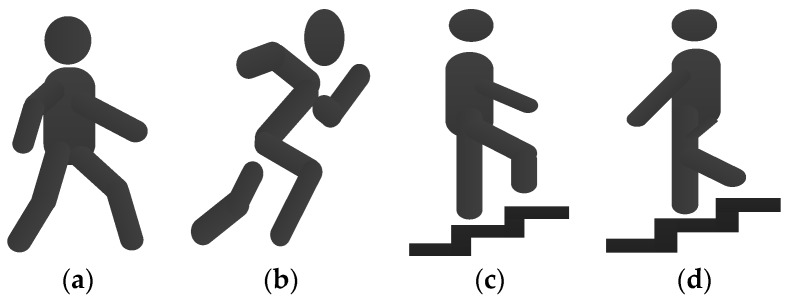
Four movement states. (**a**) Walking; (**b**) Running; (**c**) Upstairs; (**d**) Downstairs.

**Figure 6:**
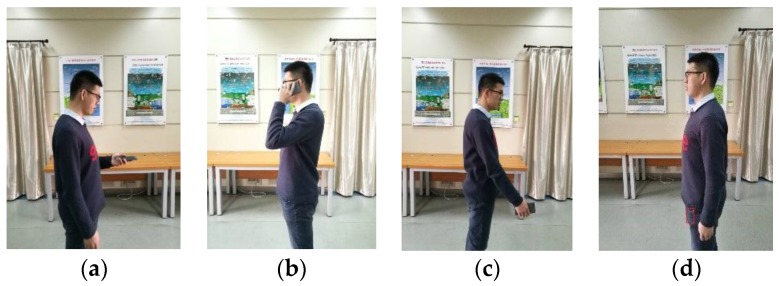
Four phone poses. (**a**) Holding; (**b**) Calling; (**c**) Swinging; (**d**) Pocket.

According to the daily movement of pedestrian and the custom of using a phone, we define the motion mode as two categories: the movement state and the phone pose. The movement state represents the global motion of pedestrian, including Walking, Running, Upstairs and Downstairs. The phone pose represents the pose of holding or placing a phone, including Holding, Calling, Swinging and Pocket, as shown in Figure 5 and Figure 6. The movement state and the phone pose are independent with each other, they can produce 16 combination modes.

The phone poses are described in detail as follows:

1) Holding: the case that the phone is held in front of the body. In this case, the phone is stable relative to the body, and the direction of the phone represents the direction of pedestrian motion, for example, the pedestrian reads a message or watches the navigation interface while walking.
2) Calling: the case that the pedestrian makes a call. In this case, the phone screen points to the side of the body.
3) Swinging: the case that pedestrian swings the phone with the hand. In this case, according to the habits of using a phone, we assume the phone screen points to the side of the body, and the phone approximately points to the direction of pedestrian motion.
4) Pocket: the case that the phone is carried in the front pocket of the trousers. In this case, we define the phone plane is approximately perpendicular to the ground when pedestrian is in static state.

### 5.3. Feature Extraction

The filtered data are insufficient to distinguish one motion mode from another, we need to extract feature data from the filtered accelerations and angular velocities in a sliding window. The size of the sliding window is 256 samples with 50% overlap and the sensor sampling frequency $f_{s}$ is 100 Hz. The magnitudes of acceleration and angular velocity signals are first calculated as:

(1) $$a_{mag}=\sqrt{a_{x}^{2}+a_{y}^{2}+a_{z}^{2}}$$

(2) $$\omega_{mag}=\sqrt{\omega_{x}^{2}+\omega_{y}^{2}+\omega_{z}^{2}}$$

where $a_{x}$, $a_{y}$ and $a_{z}$ are the measurements from 3-axis accelerometer, $\omega_{x}$, $\omega_{y}$ and $\omega_{z}$ are the measurements from 3-axis gyroscope.

The statistical features of signal include mean value and variance, which describe the average value and amplitude change of accelerations and angular velocities in a motion period. The signal energy describes the overall strength and the transmission ability of the signal. In prior works, the features are extracted from the magnitudes of the signals, however we find that the signals on each axis can better reflect different motions, especially for distinguishing different phone poses. In this paper, the time-domain feature vector $f_{1}=[M_{a},E_{a},V_{a},M_{\omega },E_{\omega },V_{\omega }]$ includes mean values, variances and energies from 3-axis signals and the magnitudes of accelerations and angular velocities. The components of $f_{1}$ are listed as follows:

- The acceleration mean value $M_{a}=[m_{ax},m_{ay},m_{az},m_{a}]$,
- The angular velocity mean value $M_{\omega }=[m_{\omega x},m_{\omega y},m_{\omega z},m_{\omega }]$,
- The acceleration variance $V_{a}=[v_{ax},v_{ay},v_{az},v_{a}]$,
- The angular velocity variance $V_{\omega }=[v_{\omega x},v_{\omega y},v_{\omega z},v_{\omega }]$,
- The acceleration energy $E_{a}=[e_{ax},e_{ay},e_{az},e_{a}]$,
- The angular velocity energy $E_{a}=[e_{\omega x},e_{\omega y},e_{\omega z},e_{\omega }]$.

**Figure 7:**
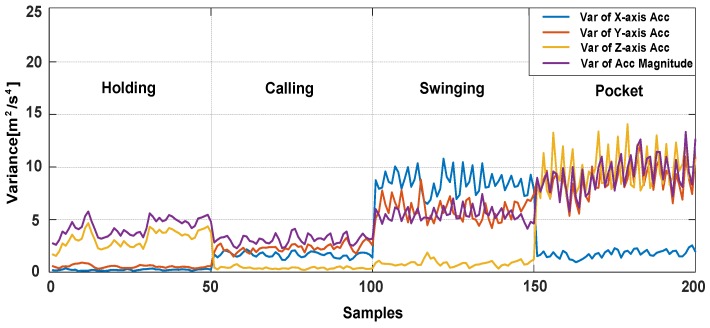
The variances of accelerations with four phone poses while pedestrian is walking.

**Figure 8:**
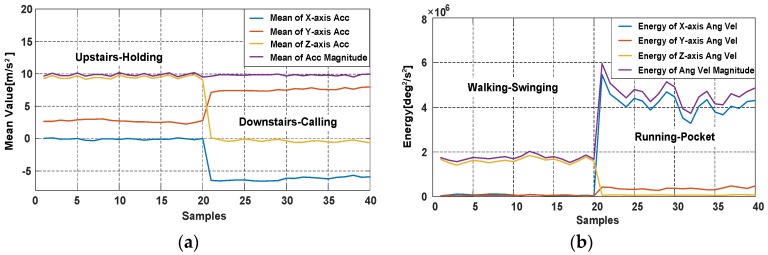
The mean values and energy of accelerometer and gyroscope data in different movement states and phone pose. (**a**) The mean values of accelerations with Holding and Calling in Upstairs and Downstairs; (**b**) The energies of angular velocities with Swinging and Pocket in Walking and Running.

The characteristics of acceleration variances with four phone poses during walking are shown in Figure 7, mean values and energies in different movement states and phone pose are also shown in Figure 8.

**Figure 9:**
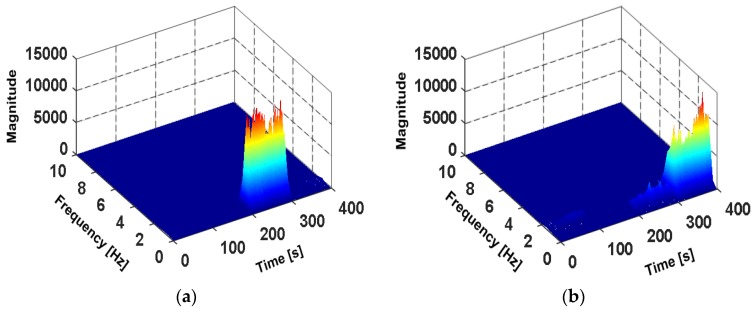

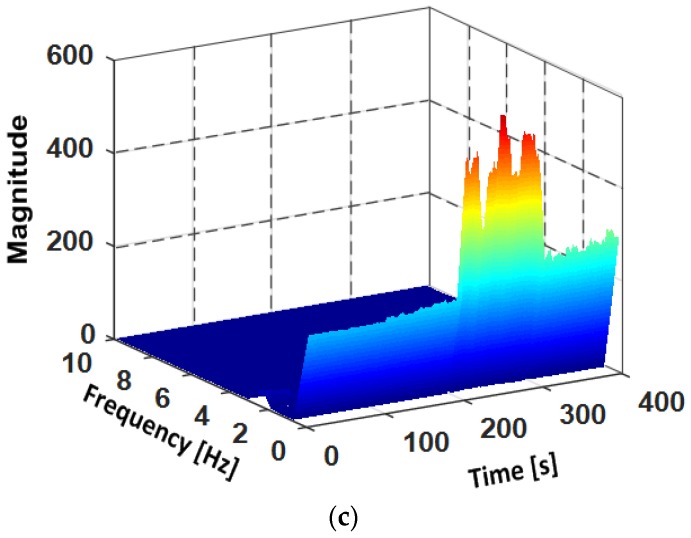
The time-frequency analysis diagram of angular velocities and accelerations in different movement states and phone poses. (**a**) The spectrogram of Z-axis angular velocities during walking; (**b**) The spectrogram of X-axis angular velocities during walking; (**c**) The spectrogram of the accelerations during running.

The time-frequency analysis of accelerations and angular velocities is performed by the Short Time Fourier Transform (STFT). In Figure 9a,b, the subject walks with four phone poses, in the order of Holding, Calling, Swinging and Pocket, the duration of each pose is 100 s. As shown in Figure 9a, it can be found that the energy spectral density of Z-axis angular velocities $s_{\omega z}$ shows significant peaks from 200 s to 300 s, this is due to the phone swings around Z-axis during the swinging of the hand. When the phone is carried in pocket, the phone is fixed relative to the thigh and rotates with the thigh. The energy spectral density of X-axis angular velocities $s_{\omega x}$ shows significant peaks from 300 s to 400 s in Figure 9b. As shown in Figure 9c, the subject runs with four phone poses, the energy spectral of accelerations *s_a_* of Swinging shows more obvious peaks than other poses. The frequency-domain feature vector is expressed as $S=[s_{a},s_{\omega x},s_{\omega z}]$.

### 5.4. Classifier

In this paper, the SVM and the DT are employed for the combination mode classification. SVM is a supervised learning model for classification, and it has the ability of processing nonlinear relations. DT is a non-parametric classifier with a tree structure, which can reflect the characteristics of the data directly. If an observation is given, the corresponding logical expression is easily introduced according to the generated DT model.

**Figure 10:**
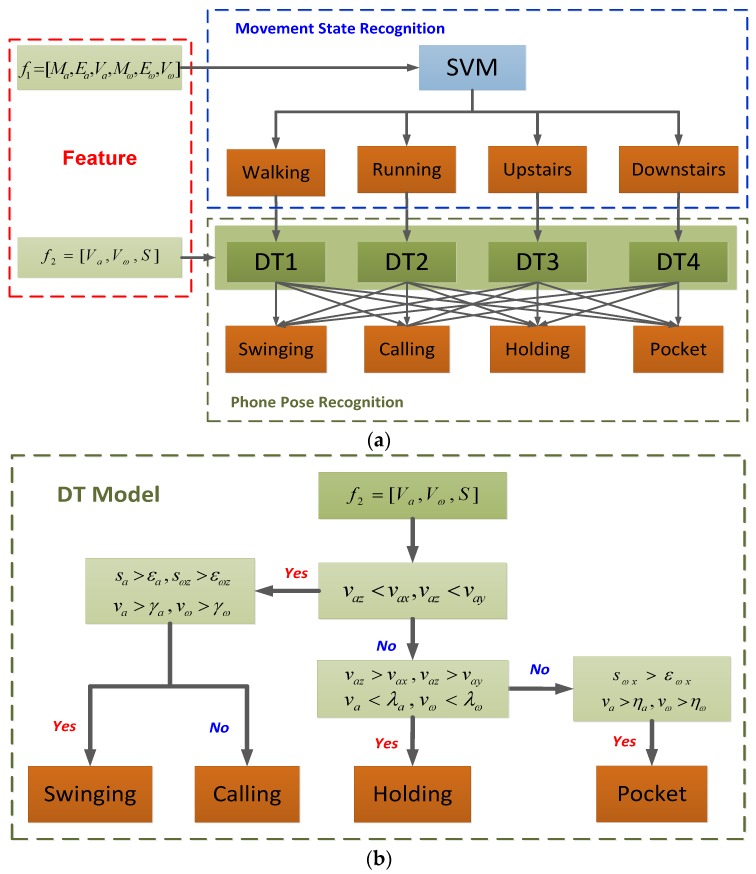
The overview of the classifier. (**a**) The scheme of movement states and phone poses recognition; (**b**) DT model for phone pose classification.

The combinations of movement state and phone pose make it very difficult to separate different movement states linearly. Thus, we utilize SVM to recognize the movement states of pedestrian. The time-domain feature vector $f_{1}=[M_{a},E_{a},V_{a},M_{\omega },E_{\omega },V_{\omega }]$ is chosen as the input vector of SVM and the output is the current movement state. In the previous sections, we have analyzed the characteristic of feature data in different phone poses. Thus, DT is utilized to recognize the phone poses according to the output of SVM. There are four DT models (DT1, DT2, DT3 and DT4), corresponding to the four SVM outputs (Walking, Running, Upstairs and Downstairs). For the SVM outputs, the corresponding DTs are selected. Figure 10a shows the architecture of movement state and phone pose recognition system. The feature vector $f_{2}=[V_{a},V_{\omega },S]$ consists of the frequency-domain feature and the variances of accelerometer and gyroscope. $f_{2}$ is the input vector of DT, and the output is the current pose of the smartphone. All feature data are divided into two groups, one group is training dataset for training the classifier parameters and the other group is testing dataset for verifying the recognition accuracy of the trained classifier. $\varepsilon_{a},\;\varepsilon_{\omega z},\;\lambda_{a},\;\lambda_{\omega },\;\eta_{a}$ and $\eta_{\omega }$ are the parameters of the DT model, they are adjusted according to the corresponding movement state. The DT model is shown in Figure 10b. In this way, the combination modes between movement states and phone poses can be recognized by the designed classifier.

## 6. Pedestrian Dead Reckoning

The data from accelerometers and gyroscopes show various characteristics in different movement states and phone poses. Therefore, based on the results of the classification, a flexible PDR algorithm for multi-motion modes is proposed, including step detection, stride length estimation and heading determination. The equation of the PDR system is:

(3) $$\{\begin{array}{c} X_{k}=X_{k-1}+L_{k-1,k}\cdot \sin(\theta_{k-1,k})\\ Y_{k}=Y_{k-1}+L_{k-1,k}\cdot \cos(\theta_{k-1,k}) \end{array}$$

where *X* and *Y* are the coordinates in the east and north, *L* is the stride length, θ is the heading angle during one stride interval, and *k* denotes the index of pedestrian’s strides.

### 6.1. Step Detection

**Figure 11:**
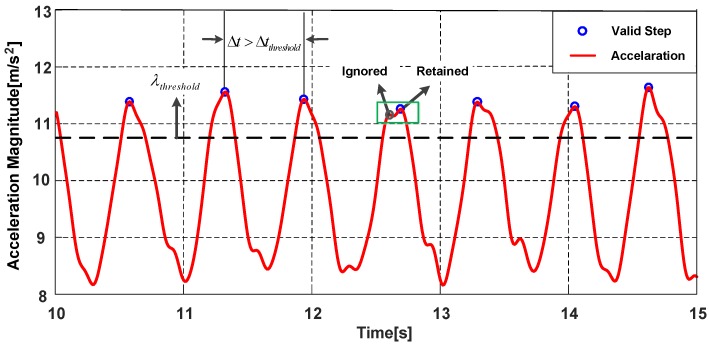
Detection of the valid steps.

The phone produces a periodic motion with the steps while a pedestrian is walking, and the accelerometer data can reflect the step characteristics. The change of device’s attitude will influence the values of the acceleration on three axes, to avoid the influence, the magnitude of the acceleration $a_{mag}$ in Equation (1) is chosen as the norm for step detection. The magnitudes of accelerations in Holding are shown in Figure 11, the acceleration magnitude presents a sinusoidal wave and the peaks represent the probable steps of pedestrian. For different movement states and phone poses, the accelerations are also different. To detect the steps accurately, the parameters of algorithm are adjusted for different movement states and phone poses, the step detection algorithm in this paper consists of three modules:

1. Candidate peaks detection

The core of the step detection algorithm is to find the peaks that can represent the actual steps of pedestrian. Thus, a candidate peak threshold $\lambda_{threshold}$ is needed to filter out the peaks caused by pedestrian irregular motions, $\lambda_{threshold}$ is obtained by analysis of the experimental data. Since the acceleration magnitude varies widely in different movement states and phone poses, the value of peak threshold is adjusted with the consideration of different motions. The peaks greater than candidate peak threshold are regarded as the candidate peaks and the others are the invalid peaks.

2. Adjacent peaks selection

Although the acceleration signals have been filtered, there are still false peaks in the filtered signal. Therefore, the adjacent peaks selection mechanism is added. We define an adjacent peaks window with a small size. If there are two or more peaks within the window, the peak with larger magnitude is retained, the peak with smaller magnitude is regarded as a false peak and ignored.

3. Step (stride) interval determination

After the above steps, we consider the time interval Δt between two consecutive candidate peaks. Pedestrians’ step frequency is less than 5 Hz [39], thus the minimum step interval threshold $\Delta t_{threshold}$ is set to 0.2 s. If the time interval Δt satisfy the threshold $\Delta t_{threshold}$, the peak is the valid peak and represents one step of the pedestrian. It should be noted that the phone presents pendulum motion in Swinging and Pocket. Therefore, each valid peak represents two steps in Swinging and Pocket, denoted as a stride. The stride interval is adopted in Swinging and Pocket, the size of the minimum stride interval threshold is two times that of the minimum step interval threshold.

### 6.2. Stride Length Estimation

The stride length varies from person to person, it should be a variable which is related to the pedestrian. For different individuals, stride length will be affected by height, gender and walking speed [40]. However, for the same pedestrian, stride length is mainly related to stride frequency of the pedestrian [37]. Many estimation models have been proposed and most models are generated by using accelerometer data, including linear model, nonlinear model and artificial neural network model [41,42,43,44]. In this paper, a linear formula from [44] is adopted to estimate pedestrian’s stride length, which is denoted as:

(4) $$L=A\cdot f_{stride}+B\cdot \sigma^{2}{}_{a}+C$$

where *L* is the stride length to be estimated. $f_{stride}$ is the stride frequency, which is the reciprocal of one stride duration and can be obtained by the step detection process. $\sigma^{2}{}_{a}$ is the variance of the accelerations during the interval of one stride. *C* is a constant, *A* and *B* are the coefficients of the stride frequency and the acceleration variance. *A*, *B* and *C* are the personalized parameters that need to be calibrated for each pedestrian. The variance $\sigma^{2}{}_{a}$ is different for different phone poses and the estimated stride length is also influenced. Thus, all model parameters are trained for each phone pose.

### 6.3. Heading Determination

Because of the characteristics of PDR, the calculation of pedestrian’s heading angles affects the precision of localization system greatly and it is also the most difficult part in the whole PDR. For Holding and Calling, the attitude of the phone is stable relative to the body while pedestrian is walking. The heading angle offset between pedestrian’s actual direction and smartphone’s direction can be easily obtained, thus pedestrian’s heading can be determined by removing the heading offset. However, for Swinging and Pocket, the attitude of smartphone changes constantly and the heading offset is not constant. As a result, the PCA-based method is used to calculate pedestrian’s headings in literatures. The PCA-based method is based on a fact that the most variations of the horizontal accelerations are parallel to pedestrian’s direction [35,37,45], thus the first eigenvector is regarded as pedestrian’s direction. In the PCA-based method, the horizontal accelerations need to be first obtained by the related vertical acceleration, the vertical acceleration is obtained while pedestrian is approximately static. However, smartphone’s attitude varies constantly in Swinging and Pocket while pedestrian is walking. More importantly, the device coordinate system varies during per stride time interval. Therefore, the obtained horizontal accelerations of one stride time interval cannot be guaranteed to be completely horizontal, and the obtained pedestrian’s direction vector will be influenced by the accelerations of the vertical plane, which will cause errors in the heading estimation.

**Figure 12:**
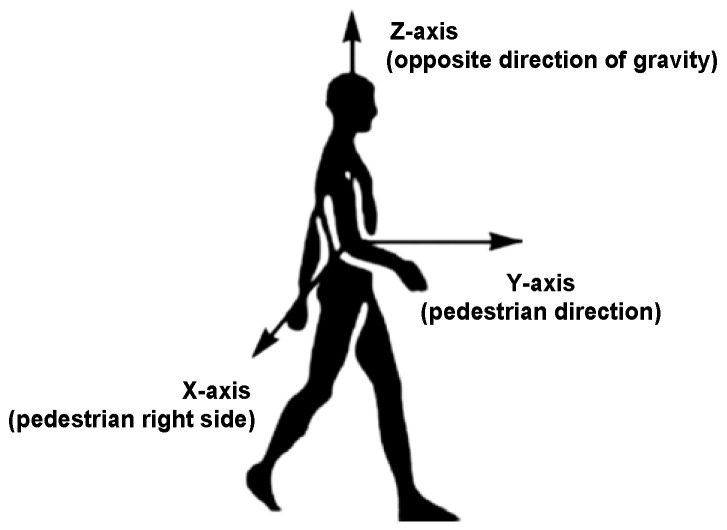
The illustration of pedestrian coordinate system.

**Figure 13:**
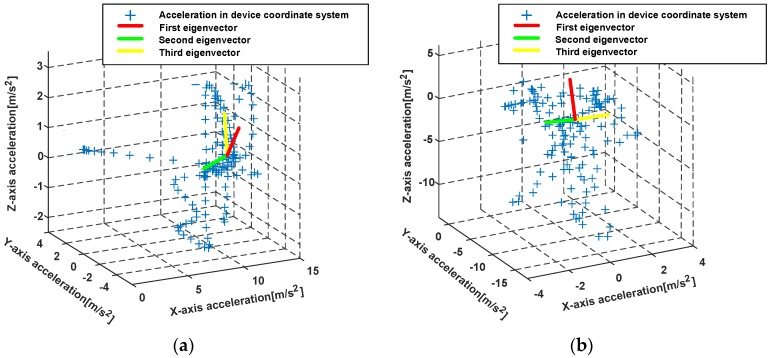
The eigenvectors extraction from the global accelerations of device coordinate system with PCA-GA. (**a**) The eigenvectors extraction for Swinging; (**b**) The eigenvectors extraction for Pocket.

To solve the mentioned problems, we present a PCA-based method with global accelerations (PCA-GA) to infer pedestrian’s headings for Pocket and Swinging. The global accelerations are the accelerations of three-axis in device coordinate system, which avoids the calculation of the horizontal accelerations. Through our observations, we found that, during walking, the position change of smartphone in X-axis of pedestrian coordinate system is minimal. The pedestrian coordinate system is depicted in Figure 12, where Y-axis points to the direction of pedestrians walking, X-axis points to the right side of pedestrian’s body and Z-axis points to opposite direction of gravity. The PCA-GA method is based on the fact that the least variations of the global accelerations are parallel to X-axis of pedestrian coordinate system. Therefore, the direction of the third eigenvector in PCA-GA is regarded as the initial right-vector $R^{*}=[r^{*}{}_{x},r^{*}{}_{y},r^{*}{}_{z}]$ of the pedestrian, the right vector is in device coordinate system and perpendicular to the pedestrian’s direction in the horizontal plane. The extracted eigenvectors from the global accelerations of device coordinate system are shown in Figure 13.

However, the right-vector $R^{*}$ acquired from PCA-GA has the problem of 180° ambiguity [35], $R^{*}$ may point to right or left side of the body. According to the definition of phone poses in Section 5.2, we analyze the orientations of three-axis of accelerometer while the smartphone is swinging in different hands. As shown in Table 1, by observing the acceleration value of X-axis (positive or negative), we can easily know which hand is holding the phone. Take the left-hand case as an example, Z-axis of device coordinate system is approximately to the right side of the body, thus the component $r_{z}$ of right-vector $R^{*}$ should be positive. If this is not satisfied, we take the opposite vector of right-vector as actual right-vector $R=[r_{x},r_{y},r_{z}]$.

**Figure 14:**
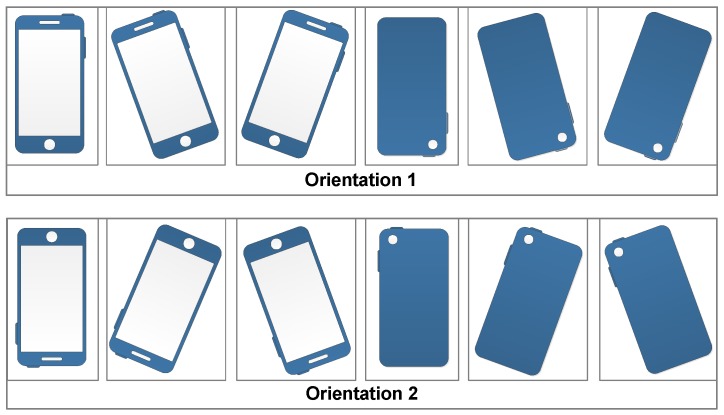
Different orientations of the smartphone in the pocket.

According to the definition of Pocket, the phone screen plane is approximately vertical to the ground. When the smartphone is carried in the pocket of trousers, we divide its orientations into two major categories, as shown in Figure 14. O1 and O2 represent the smartphone’s X-axis points to right side and left side of pedestrian, respectively.

**Figure 15:**
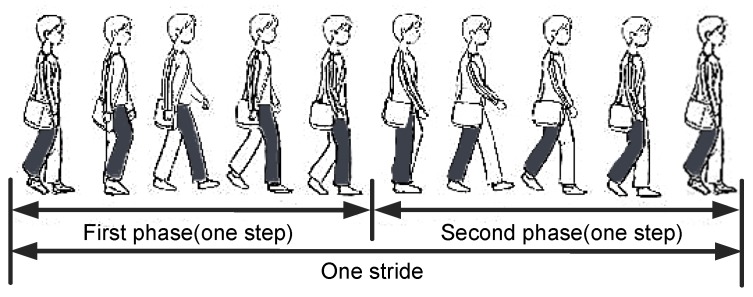
The illustration of pedestrian’s one stride.

**Figure 16:**
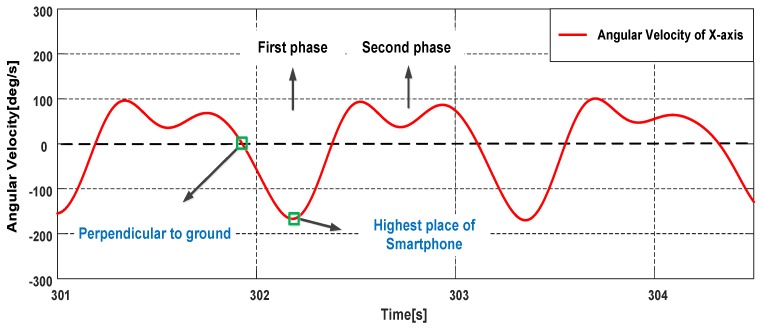
The angular velocities on X-axis while the smartphone is carried in the pocket.

Pedestrian’s one stride can be divided into two phases as shown in Figure 15, we describe the movement process in the case of the phone in the right pocket of trousers. Looking to the right side of the body, during the first phase: the right thigh does the counterclockwise motion to a certain height first, then begins to do the clockwise motion; during the second phase: the right thigh continue the clockwise motion until the right foot leaves the ground, then does the counterclockwise motion. The phone’s rotation generates angular velocities on X-axis of gyroscope during one stride. Take O1 as an example, looking to the right side of the body, in the first (second) phase, the angular velocities on X-axis are positive (negative) with the counterclockwise (clockwise) rotation. Because the thigh has a lifting motion in the first phase, the variation range of the angular velocities on X-axis in the first phase is greater. Therefore, we can infer the smartphone’s orientation by the angular velocities on X-axis. As shown in Figure 16, the absolute value of the trough is greater than the peak in a stride period, thus the smartphone’s orientations belong to O2. In this case, the component $r_{x}$ of the right-vector $R^{*}$ should be negative, if this is not satisfied, we take the opposite vector of the right-vector as the actual right-vector $R=[r_{x},r_{y},r_{z}]$.

The heading angle is calculated by the right vector R and the east vector E (mentioned in the following), we first project the right-vector and the magnetic vector onto the horizontal plane. It is realized by the estimated gravity vector [46], and the estimated gravity vector is:

(5) $$G=-(g_{x},g_{y},g_{z})$$

where $g_{x}$, $g_{y}$ and $g_{z}$ are the averages of all the measurements on the respective axis in the time interval of one stride.

The right-vector is denoted as:

(6) $$R=(r_{x},r_{y},r_{z})$$

where $r_{x}$, $r_{y}$ and $r_{z}$ represent the component on the respective axis of device coordinate system.

Then, vector dot product is used to calculate the projection $R_{v}$ of R upon the estimated gravity vector G, in other words, $R_{v}$ is the component of right vector in the vertical plane:

(7) $$R_{v}=\left(\frac{R\cdot G}{G\cdot G}\right)G$$

The horizontal component $R_{h}$ can be obtained by the vector subtraction:

(8) $$R_{h}=R-R_{v}$$

The estimated gravity vector G points to the downward and the magnetic vector M points to the north and downward in the northern hemisphere. The east vector E is perpendicular to both G and M, thus the east vector can be obtained by vector cross product [47] in the device coordinate system. The magnetic vector is approximated by averaging the readings of magnetometer in the time interval of one stride:

(9) $$M=(m_{x},m_{y},m_{z})$$

(10) E=G×M

where $m_{x}$, $m_{y}$ and $m_{z}$ are averages of all the measurements on the respective axis in the time interval of one stride.

**Figure 17:**
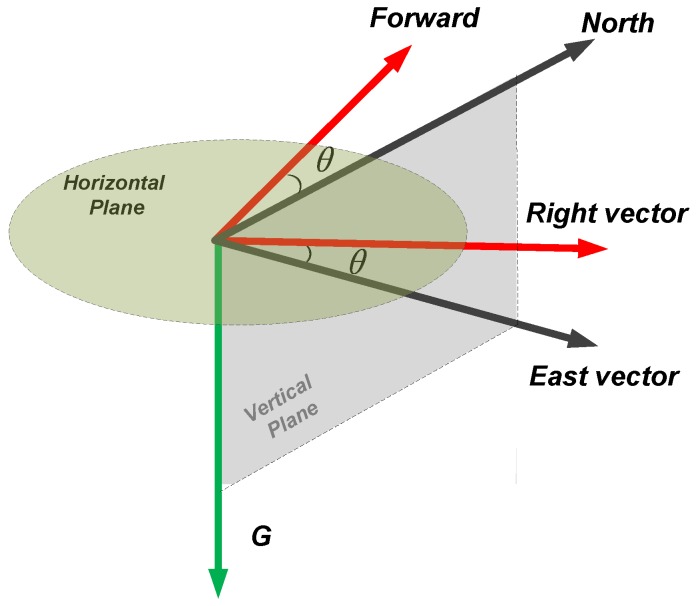
The illustration of heading angle determination by east vector and right vector.

As shown in Figure 17, the red arrows represent the forward and right direction of pedestrian, the black arrows represent the horizontal component of the magnetic vector and the east vector, and the green arrow represents the estimated gravity vector. Obviously, the heading angle is the angle between the east vector and the right vector, which is obtained by vector dot product:

(11) $$\theta =\arccos(\frac{R_{h}\cdot E}{\left|R_{h}\right|\left|E\right|})$$

where the obtained heading angles are within the interval from 0 to π, and they are needed to be extended to the interval from 0 to 2π, it can be solved by vector cross product:

(12) $$\theta =\{\begin{array}{ll} \theta -\beta , & (E\times R_{h})\cdot G>0\\ 2\pi -\theta -\beta , & (E\times R_{h})\cdot G<0 \end{array}$$

where the vector cross product $E\times R_{h}$ is in the same (opposite) direction as the gravity vector, when the right vector is on the right (left) side of the east vector, β is the local magnetic declination.

## 7. Experiments and Results

In this section, the experiments are presented to verify the performance of the proposed methods of motion mode recognition and indoor localization. The experimental equipment is HUAWEI honor 8 smartphone, the sensor sampling frequency is 100 Hz, the sensor signals are transmitted to the computer by a wireless network in real time.

### 7.1. Motion Mode Recognition Experiment

We selected five males and five females to test the motion mode recognition performance. The participants move with different movement states and phone poses, and the start and end of each mode are marked. The total data collection duration of each participant is about 15 min, including 5 min for walking, 3 min for running, 3.5 min for upstairs and 3.5 min for downstairs. The size of the sliding window for feature extraction is set to 256 samples with 50% overlap. Thus, the instances of motion modes are equal to the data collection duration divided by 1.28 s. In this experiment, 60% of the feature data are chosen for training dataset and 40% are chosen for testing dataset. The instances for testing are listed in Table 2.

The global confusion matrix for movement states and phone poses recognition from the classifier is listed in Table 3, the rows represent the instances of actual motion modes and the columns represent the instances of motion modes recognized by the classifier. To describe the results more clearly, the confusion matrixes for movement states and phone poses are listed respectively, as shown in Table 4 and Table 5.

It can be seen from Table 3 that the average recognition accuracy of the combination modes of movement states and phone poses are 92.4%. As shown in Table 4, the overall recognition accuracy of movement states is over 94.1%, and the recognition accuracy of Walking can be to 97.5%. 1.6% instances of Walking are misrecognized as Running and 2.3% instances of Running are misrecognized as Walking. There is about 3% misrecognition between Upstairs and Downstairs, that is because the two states have many similar characteristics, the problem can be solved by adding other sensors, such as the barometer.

**Figure 18:**
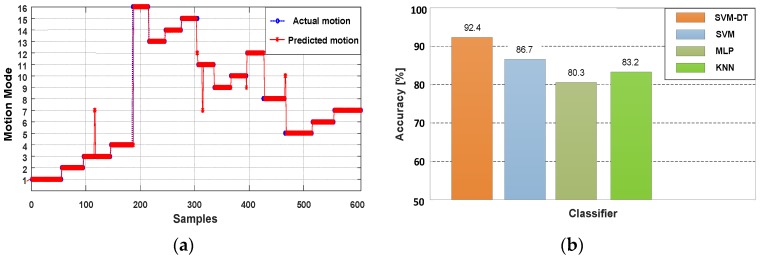
Performance of recognizing the combination modes of movement states and the phone poses. (**a**) Combination mode predictions with SVM-DT; (**b**) Recognition accuracy comparison of SVM-DT, SVM, MLP and KNN.

It can be seen from Table 5 that the overall recognition accuracy of the phone poses is over 93.8%, and the recognition accuracy of Swinging can be to 97.1%. A few (3.4%) instances of Calling are misrecognized as Holding. For the designed classifier, the movement states are determined first, and the results of SVM will make an influence on the next classification stage. Therefore, although the recognition of Swinging is the best, there are still 2.0% instances regarded as Pocket. The recognition result of all 16 combination modes is shown in Figure 18a, where motions (1–4), (5–8), (9–12) and (13–16) represent Walking, Downstairs, Upstairs and Running respectively, the phone poses are in the order of Holding, Calling, Swinging and Pocket in the duration of each movement state. To evaluate the performance of the proposed classifier, we compare proposed classifier, SVM, multilayer perceptron (MLP) and k-nearest neighbor (KNN) to recognize the combination modes of movement states and phone poses. As shown in Figure 18b, compared with the other classifiers, our proposed classifier can achieve better performance.

### 7.2. Localization Experiment

We select two participants to verify the performance of the step detection algorithm. The participants walk along a straight and flat road, the walking distance of each phone pose is about 700 m. For Swinging and Pocket, the steps are two times the detected strides, the results are listed in Table 6.

As shown in Table 6, the detection accuracy for Holding and Calling are both more than 99%. For Swing and Pocket, once one stride is not detected, it means two steps are missed. Thus, compared with Holding and Calling, the misdetection is higher, for all that, the accuracy can still be more than 98.4%. From the results of the experiment we can see that, with the mechanism of adjacent peaks selection and step interval decision, the accuracy of step detection can be guaranteed.

**Figure 19:**
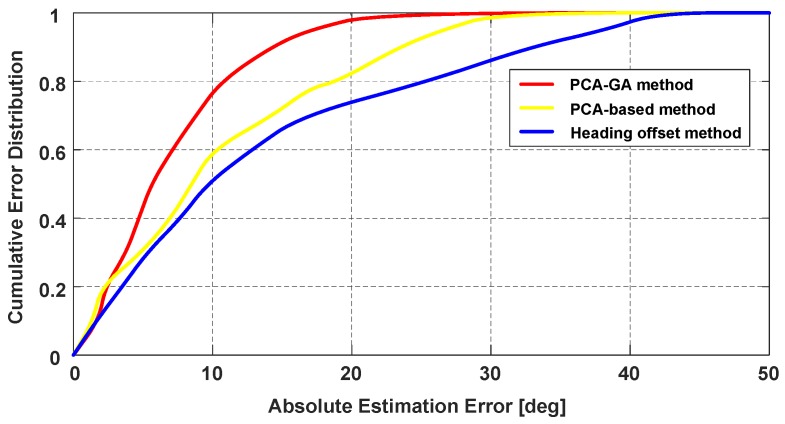
Performance comparison of different heading estimation methods (smartphone in a pocket).

To evaluate the performance of the proposed heading determination method, we compare PCA-GA with the traditional PCA-based method and the heading offset estimation method from [27]. In the comparison experiment, the smartphone is carried in the pocket. As shown in Figure 19, the 50% absolute estimation errors of heading for PCA-GA, PCA-based and heading offset method are $5.6^{\circ }$, $8.5^{\circ }$ and $9.8^{\circ }$, respectively. The 75% absolute estimation errors of heading are $9.2^{\circ }$, $16.1^{\circ }$ and $21.2^{\circ }$, respectively. As mentioned in the previous section, the PCA-based method requires the calculation of the horizontal component of accelerations, thus the extracted forward vector can be influenced by the change of smartphone’s attitude. Besides, heading offset cannot remain constant when the pedestrian puts the smartphone in the pocket. Since the improved PCA-GA method avoids these problems, the PCA-GA method can obtain higher accuracy.

**Figure 20:**
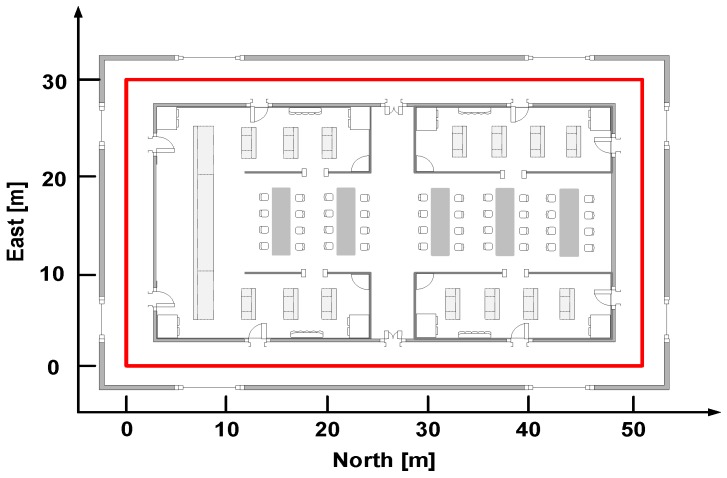
Indoor localization experiment environment.

The localization experimental site is situated at an office building, as shown in Figure 20, which can be regard as an approximate laboratory environment and this is beneficial to the heading estimation with the magnetometer. The red line represents the pedestrian’s trajectory and the total length is about 164 m. We place the markers on the path, the participant walks and runs along the marked path with four phone poses respectively, the walking and running speeds are about 1.3 m/s and 2.5 m/s. The localization experiments are executed on the horizontal plane without considering the change of pedestrian’s height.

**Figure 21:**
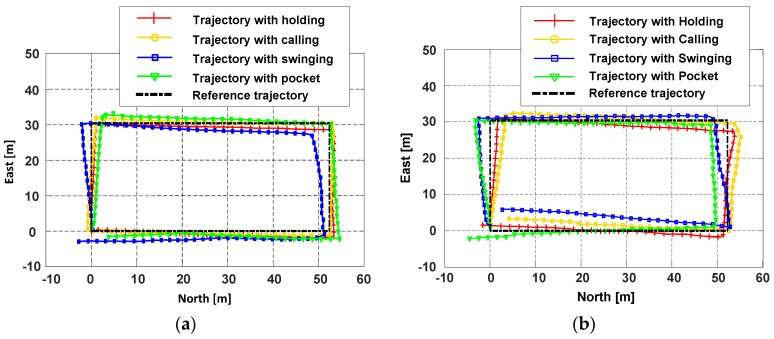
The estimated trajectories of the participant with four phone poses during walking and running. (**a**) The estimated trajectories of four phone poses during walking; (**b**) The estimated trajectories of four phone poses during running.

**Figure 22:**
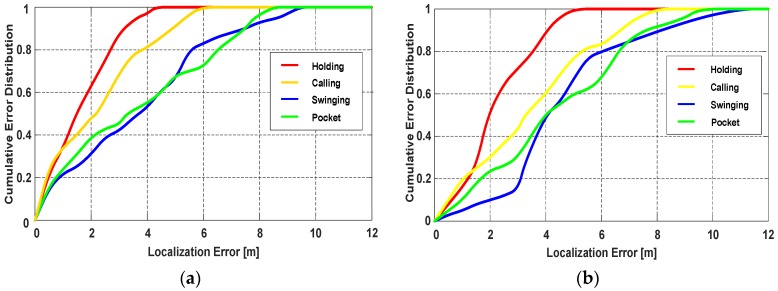
The localization performance of the participant with four phone poses during walking and running. (**a**) The cumulative error distribution of four phone poses during walking; (**b**) The cumulative error distribution of four phone poses during running.

Figure 21a,b present the participant’s trajectories during walking and running, respectively. The cumulative distribution of the localization errors during walking is shown in Figure 22a, since the attitude of phone is more stable, the localization performance of Holding and Calling is the best, and the mean error can be within 2 m. For Swinging and Pocket, the participant holds the smartphone more freely, due to the influence of the dynamic change of smartphone, the mean error reduces to about 3 m. Since running will inevitably cause the body to shake, the heading angle offset during running is not as stable as walking. Besides, the shake of the body will have a slight effect on the right vector extraction of PCA-GA. Thus, as shown in Figure 22b, the localization errors of Running are larger than that of Walking. Table 7 lists the localization errors in detail, when the smartphone is in the pocket or swinging and the pedestrian is walking or running, the proposed PDR can achieve around 3.5 m mean error in a trajectory of 164 m.

## 8. Conclusions and Future Work

This paper presents an indoor localization method based on motion mode recognition. First, the SVM and the DT are utilized, and all 16 combination modes of four movement states and four phone poses can be recognized accurately. Then, the parameters of step detection and stride length estimation are trained and adjusted depends on various movement states and phone poses. Finally, we improve the traditional PCA-based method by developing the PCA-GA-based method to infer pedestrians’ headings. Besides, we further analyze the orientations of the smartphone carried in the front pocket of trousers, and consider whether the smartphone is in pedestrian’s left hand or right hand. By analyzing the data of accelerometer and gyroscope, the ambiguity of extracted right vector is eliminated. The experiment results show that the average recognition accuracy of the combination modes of movement states and phone poses is 92.4%. The 50% and 75% absolute errors of heading estimation with PCA-GA are $5.6^{\circ }$ and $9.2^{\circ }$, respectively. PCA-GA method can obtain higher accuracy than the traditional PCA-based method and heading offset method. The localization experiments indicate that the proposed PDR can reduce the mean error to around 3.5 m in a trajectory of 164 m, in the case that pedestrians moving in different states and the smartphone is held in different poses. For future work, we plan to integrate more smartphone sensors to improve the performance of the indoor localization system, and a more detailed classification of the locomotion activity can be conducted.

## Figures and Tables

**Table 1:** The approximate orientations of accelerometer three-axis while smartphone is swinging in left or right hand.

Phone Pose	Hand	X-Axis	Y-Axis	Z-Axis
Swinging	left-hand	direction of gravity	Pedestrian’s direction	Pedestrian’s right side
	right-hand	opposite direction of gravity	Pedestrian’s direction	Pedestrian’s left side

**Table 2:** The instances of motion modes for testing.

	Holding	Calling	Swinging	Pocket
Walking	250	250	250	250
Running	140	140	140	140
Upstairs	160	160	160	160
Downstairs	160	160	160	160

**Table 3:** The global confusion matrix for movement states and phone poses recognition.

		Walking				Running				Upstairs				Downstairs				
		H	C	S	P	H	C	S	P	H	C	S	P	H	C	S	P	
Walking	Holding	**238**	4	0	3	2	1	0	2	0	0	0	0	0	0	0	0	**95.2%**
	Calling	7	**232**	3	2	2	4	0	0	0	0	0	0	0	0	0	0	**92.8%**
	Swinging	0	0	**242**	2	0	0	3	0	0	0	0	0	0	0	1	2	**96.8%**
	Pocket	0	0	9	**233**	0	0	0	2	0	0	2	4	0	0	0	0	**93.2%**
Running	Holding	1	0	1	3	1**27**	3	0	0	1	0	0	0	3	1	0	0	**90.7%**
	Calling	0	2	0	4	3	**125**	1	0	0	0	0	0	3	2	0	0	**89.3%**
	Swinging	0	0	1	0	0	3	**134**	2	0	0	0	0	0	0	0	0	**95.7%**
	Pocket	0	0	0	1	1	1	4	**129**	0	0	0	2	0	0	0	2	**92.1%**
Upstairs	Holding	1	2	0	0	0	0	0	0	**150**	2	0	0	5	0	0	0	**93.8%**
	Calling	1	2	0	0	0	0	0	0	3	**150**	0	0	2	2	0	0	**93.8%**
	Swinging	0	0	0	0	0	0	2	0	1	0	**152**	2	0	1	2	0	**95.0%**
	Pocket	1	0	0	1	0	0	0	3	0	2	5	**141**	0	0	1	6	**88.1%**
Downstairs	Holding	2	0	0	0	2	0	0	0	7	0	0	0	**144**	2	0	3	**90.0%**
	Calling	0	0	0	0	0	4	0	0	0	3	0	7	3	**140**	0	3	**87.5%**
	Swinging	0	0	0	0	0	0	3	0	0	0	2	1	0	0	**151**	3	**94.4%**
	Pocket	0	0	0	2	1	0	0	3	0	0	0	1	3	0	5	**145**	**90.6%**
		**97.5%**				**94.5%**				**95.0%**				**94.1%**				

**Table 4:** Confusion matrix for movement states recognition.

	Walking	Running	Upstairs	Downstairs
Walking	**97.5%**	1.6%	0.6%	0.3%
Running	2.3%	**94.5%**	0.5%	2.7%
Upstairs	1.3%	0.8%	**95.0%**	2.9%
Downstairs	0.6%	2.0%	3.3%	**94.1%**

**Table 5:** Confusion matrix for phone poses recognition.

	Holding	Calling	Swinging	Pocket
Holding	**96.2%**	2.1%	0.1%	1.6%
Calling	3.4%	**93.8%**	0.6%	2.2%
Swinging	0.1%	0.8%	**97.1%**	2.0%
Pocket	0.8%	0.4%	3.7%	**95.1%**

**Table 6:** The accuracy of the proposed step detection algorithm.

Phone Poses	Actual Steps	Detected Steps	Accuracy
Holding	1034	1029	99.5%
Calling	1020	1012	99.2%
Swinging	983	971	98.8%
Pocket	974	958	98.4%

**Table 7:** The localization errors with four phone poses during walking and running.

Phone Pose	Localization Errors of Walking (m)			Localization Errors of Running (m)		
	Mean Error	50% Error	95% Error	Mean Error	50% Error	95% Error
Holding	1.38	1.47	3.62	1.79	1.92	4.39
Calling	2.06	2.23	5.35	2.88	3.24	7.16
Swinging	3.38	3.72	8.69	3.95	3.93	9.22
Pocket	3.05	3.26	7.80	3.66	4.08	8.91
